# Total hydrocortisone dosage in extremely low birth weight infants and neurodevelopment up to school age

**DOI:** 10.1038/s41390-025-04426-x

**Published:** 2025-09-23

**Authors:** Akinobu Taniguchi, Basile Chrétien, Takashi Maeda, Kazuto Ueda, Ryosuke Miura, Ryuichi Tanaka, Toshihiko Suzuki, Yukako Muramatsu, Erina Kataoka, Eiko Kato, Hikaru Yamamoto, Koji Takemoto, Miharu Ito, Seiji Hayashi, Yuichiro Sugiyama, Kazuki Nishida, Yoshiaki Sato

**Affiliations:** 1 Division of Neonatology, Center for Maternal-Neonatal Care, Nagoya University Hospital, Nagoya, Japan; 2 Biostatistics Section, Department of Advanced Medicine, Nagoya University Hospital, Nagoya, Japan; 3 Department of Pediatrics, Anjo Kosei Hospital, Anjo, Japan; 4 Department of Pediatrics, Tosei General Hospital, Seto, Japan; 5 Department of Neonatology, Toyota Memorial Hospital, Toyota, Japan; 6 Department of Pediatrics, Konan Kosei Hospital, Konan, Japan; 7 Department of Pediatrics, Ogaki Municipal Hospital, Ogaki, Japan; 8 Department of Pediatrics, Okazaki City Hospital, Okazaki, Japan; 9 Department of Pediatrics, Japanese Red Cross Aichi Medical Center Nagoya Daiichi Hospital, Nagoya, Japan

## Abstract

**Background:**

An association between total hydrocortisone (HC) dosage in infants with extremely low birth weight (ELBW) and subsequent neurodevelopmental outcomes up to school age remains unclear.

**Method:**

We conducted a retrospective longitudinal cohort study across eight centers in Japan, including ELBW infants born between 2015 and 2017. We investigated the association between total HC dosage administered up to 36 weeks postmenstrual age and neurodevelopmental outcomes to school age.

**Results:**

Linear mixed model analysis showed a significant association between higher HC dosage and lower developmental and intelligence quotient (DQ/IQ) scores. This trend persisted at 6 years of age, suggesting a sustained effect of HC on cognitive outcomes. For every 10 mg increase in HC dosage, IQ scores decreased by 2.82 points (95% CI: −3.89 to −1.06, *p* = 0.001). The interaction term between HC dosage and time was not statistically significant (0.10, 95% CI: −0.18 to 0.37, *p* = 0.481), suggesting the association of HC dosage on DQ/IQ did not vary substantially throughout the study period.

**Conclusions:**

We found a relationship between total neonatal HC dosage in ELBW infants and DQ/IQ scores over time that persisted at school age. Clinicians should be aware of this potential dose-dependent effect on neurodevelopmental outcomes.

**Impact:**

- As neonatal dexamethasone administration is known to affect neurodevelopment outcomes, hydrocortisone (HC) is considered an alternative to dexamethasone as a glucocorticoid treatment.
- In infants with extremely low birth weight (ELBW), a relationship has been noted between total HC dosage and neurodevelopment in early childhood.
- We confirmed the association between total HC dosage in infants with ELBW and poor developmental and intelligence quotients to school age.
- Although HC is commonly used in the management of ELBW infants, clinicians should be aware of its potential dose-dependent effects on neurodevelopmental outcomes.

## Introduction

Infants with extremely low birth weight (ELBW), defined as those weighing <1000 g at birth, often present with unstable respiratory and circulatory status, necessitating treatment in a neonatal intensive care unit (NICU). These infants require various interventions, including postnatal steroids.^1–3^ Steroid treatments, particularly dexamethasone, have long been used to manage hypotension, including late-onset circulatory collapse (LCC),^4^ and to prevent or treat bronchopulmonary dysplasia (BPD).^5^ Dexamethasone, a potent corticosteroid, was widely used in infants in the 1990s to 2000s due to its short-term benefits in respiratory outcomes.^6,7^ However, its use has significantly declined due to mounting evidence of neurodevelopmental harm, including cognitive impairment and increased risk of neurodevelopmental disorders such as cerebral palsy.^8–10^ Even low doses of dexamethasone have been associated with poor neurodevelopment outcomes in both animal models and human studies.^11,12^ Therefore, clinical guidance now generally discourages the routine use of dexamethasone in ELBW infants unless absolutely necessary.^13^

Hydrocortisone (HC) is a less potent corticosteroid with a different pharmacokinetic profile. Therefore, it is considered a viable alternative to dexamethasone for treating ELBW infants, with initial studies suggesting that it might present fewer neurodevelopmental risks.^14,15^ In newborn rats, HC has not shown the detrimental effects of dexamethasone treatment on neurodevelopment.^15,16^ Randomized controlled trials of HC use in ELBW infants suggest fewer adverse neurodevelopmental effects compared with dexamethasone, with no significant long-term effects on cognition or motor development at follow-up ages of 2 to 5 years.^17–22^

However, while HC appears a safer option for the preservation of normal neurodevelopment, two studies in 2023 examining the effect of the total cumulative HC dosage on the subsequent development of low birth weight infants found that neonatal HC administration might be associated with neurodevelopmental delays and impairments in children up to the age of 3.^23,24^ The mechanisms underlying these effects remain unclear, and further research is needed to clarify the long-term cognitive outcomes of HC therapy in ELBW infants.

Currently, there are no reports evaluating the association between total neonatal HC dosage in ELBW infants and neurodevelopment at school age, particularly in terms of a potential dose-dependent effect. In addition, all the reports cited above have investigated the relationship between HC treatment of infants and their neurodevelopment at specific times rather than how these effects change over time. Therefore, it is unclear whether any neurodevelopmental effects, if present, diminish, stabilize, or increase as the child grows. Given the increasing use of HC as an alternative to dexamethasone, it is critical to understand the long-term cognitive consequences of HC administration in ELBW infants and changes over time in the effects of HC on neurodevelopment.

This study aimed to clarify the association between the total neonatal HC dosage in ELBW infants and their subsequent neurodevelopmental trajectory, as measured by their developmental and intelligence quotients at different time points throughout early childhood up to school age. We hypothesized that the total neonatal HC dosage would be related to neurodevelopment over time.

## Methods

### Study design and participants

This retrospective multicenter longitudinal cohort study included infants with ELBW (defined as a birth weight of <1000 g) born between April 1, 2015 and March 31, 2017 at Nagoya University Hospital, Anjo Kosei Hospital, Japanese Red Cross Aichi Medical Center Nagoya Daiichi Hospital, Konan Kosei Hospital, Ogaki Municipal Hospital, Okazaki City Hospital, Tosei General Hospital, and Toyota Memorial Hospital. Steroids are used when the neonatologist determines that they are necessary for treatment, such as to improve respiratory status requiring sustained fraction of inspiratory oxygen (FiO2) > 0.4, or to improve hypotension or circulatory failure due to LCC or infection. Maternal and neonatal information and follow-up information were collected from the institutional medical records. All participating centers used standardized definitions and harmonized data collection protocols to ensure consistency across the eight units.

### HC dosage

The total HC dose was calculated as the cumulative absolute amount (mg) from birth to 36 weeks postmenstrual age (PMA). This was defined as the total amount of intravenously or orally administered HC from birth to 36 weeks PMA. It has been reported that HC is absorbed orally almost as well as intravenously.^25^ Therefore, oral and intravenous doses were considered equivalent in this study. To improve readability, the effect estimates were presented as the change in outcome per 10 mg increase.

### Developmental and Intelligence Quotient (DQ/IQ) Measurements

The primary outcome was neurodevelopment, which was assessed at three time points: 18 months (corrected age), 3 years, and 6 years.

The results of neurodevelopmental examinations with the Kyoto Scale of Psychological Development (KSPD) (2001) at 18 months (corrected age) and 3 years were collected from medical records. The 18-month exams were performed between 18 and 24 months (corrected age), and the 3-year exams were performed between 3 and 4 years.

The KSPD was the most standardized and validated developmental test available at all the centers that participated in the follow-up study of the Neonatal Research Network in Japan. Scores on this scale correlate well with those on the Bayley scale for infant development III.^26^ The DQ was calculated by dividing the neurodevelopmental age indicated by the KSPD by the child’s chronological age corrected for prematurity and multiplying the result by 100. The protocol in Japan for the follow-up of infants with ELBW classifies an overall DQ of <70 as “delayed,” a DQ between 70–84 as “subnormal,” and a DQ ≥ 85 as “normal” neurodevelopmental function.^27^

When each child was 6 years old, the Wechsler Intelligence Scale for Children (WISC) was used to assess their IQ. This was administered as the full-scale Japanese version of either the WISC–IV or the WISC–V. The IQ score obtained was used as our measure of intellectual ability. The WISC was administered to participating children between the ages of 5 and 7 years.

### Complications and confounding factors

We collected data on complications such as sepsis, necrotizing enterocolitis (grades ≥II),^28,29^ intracranial hemorrhage (grades ≥III),^30^ cystic periventricular leukomalacia, BPD, and LCC. In this study, sepsis was defined as an organ dysfunction associated with an infection with positive blood culture. BPD was defined as the need for supplemental oxygen, positive-pressure ventilation, or both at 36 weeks PMA. LCC was diagnosed by the attending doctor according to the diagnostic criteria of the Japanese Study Group for Neonatal Endocrinology^4^ These are: (1) LCC occurring outside the transitional period; (2) A stable period before LCC onset; (3) The absence of clear causes such as sepsis, massive bleeding, or necrotizing enterocolitis before LCC onset; (4) Sudden onset hypotension and/or oliguria; (5) Hypotension and/or oliguria resistant to intravenous volume expanders and inotropes.

Because dexamethasone is known to affect neurodevelopment, cases in which dexamethasone was used were excluded from our final analysis.

The key exposure variable was the total cumulative neonatal dosage of HC. Severe complications, BPD, and LCC were included in our primary analysis as covariates to control for potential confounding factors. Severe complications were defined as the presence of any of the following: sepsis, necrotizing enterocolitis (grade ≥II), intracranial hemorrhage (grade ≥III), or cystic periventricular leukomalacia.

### Statistical analysis

Categorical variables were described as frequencies and percentages, while continuous variables were reported as medians and interquartile ranges. Preliminary analyses were performed using interaction plots to investigate heterogeneity and attrition bias over time. We also conducted a nonlinear trend assessment using locally estimated scatterplot smoothing (LOESS) to confirm the appropriateness of modeling linear IQ trajectories.

For the main analyses, a linear mixed model (LMM) was used to evaluate the longitudinal relationship between total neonatal HC dosage and DQ/IQ scores. The model variables included time, BPD, LCC, severe complications and the interaction between HC dosage and time. A random intercept was specified for each participant to account for between-subject variability. The adjusted model was expressed as follows:

$${{{\rm{DQ}}}}/{{{\rm{IQ}}}}\_{{{\rm{ij}}}}= \,	{{{\rm{\beta }}}}0+{{{\rm{\beta }}}}1\times ({{{\rm{HC}}}}\; {{{\rm{dosage}}}}\; {{{\rm{per}}}}10{{{\rm{mg}}}})\_{{{\rm{ij}}}}+{{{\rm{\beta }}}}2\times ({{{\rm{time}}}}\; {{{\rm{point}}}})\_{{{\rm{ij}}}}\\ 	+{{{\rm{\beta }}}}3\times ({{{\rm{severe}}}}\; {{{\rm{complication}}}})\_{{{\rm{ij}}}}+{{{\rm{\beta }}}}4\times ({{{\rm{BPD}}}})\_{{{\rm{ij}}}}+{{{\rm{\beta }}}}5\times ({{{\rm{LCC}}}})\_{{{\rm{ij}}}}\\ 	+{{{\rm{\beta }}}}6\times ({{{\rm{HC}}}}\; {{{\rm{dosage}}}}\times {{{\rm{time}}}}\; {{{\rm{point}}}})\_{{{\rm{ij}}}}+{{{\rm{u}}}}0{{{\rm{j}}}}+{{{\rm{\varepsilon }}}}\_{{{\rm{ij}}}}$$

Where:

- DQ/IQ_ij denotes the cognitive score for the i-th measurement occasion of the j-th participant.
- β0 represents the intercept.
- β1 through β6 represent the fixed effects of HC dosage, time, severe complications, BPD, LCC, and their interactions, respectively.

- u0j represents the random intercept for the j-th participant, capturing individual differences between the participants. These random intercepts were assumed to have a normal distribution with a mean of 0 and variance of τ_00_.
- ε_ij represents the residual error term, accounting for variance in cognitive scores not explained by the model, including the effects of other factors and measurement errors. This residual was also assumed to have a normal distribution, with a mean of 0 and variance of σ².

Due to the limited sample size and risk of overfitting, it was not feasible to include all potential covariates simultaneously in a single model. Therefore, we constructed a primary adjusted model including severe complications, BPD, and LCC. Additionally, we performed sensitivity analyses by substituting birth weight or gestational weeks for the “severe complications” variable to examine the robustness of the association between HC dosage and neurodevelopmental outcomes. Missing data patterns were examined and categorized by reasons, and a supplemental table was created to compare characteristics between individuals with complete and incomplete data, enabling the assessment of potential biases. All statistical analyses were performed using R software, v. 4.4.2. The two-sided significance level was set as 5% (*p* < 0.05).

## Results

### Overall results

Table 1 summarizes the basic demographic and clinical characteristics of the participants. The cohort analyzed in this study was composed of 218 ELBW infants born at the participating hospitals. During NICU hospitalization, 20 of these infants died. Of the 198 surviving patients who were discharged from the NICU, 135 (68%) were followed up until the age of 6 years (Supplemental Table S1 and Supplemental Fig. S1). The developmental or intellectual tests were performed in 159, 144, and 100 patients at 18 months (corrected age), 3 years, and 6 years, respectively. HC was administered to 109 (55%) of the 198 surviving patients during their time in the NICU. The mean DQ/IQ scores were 88 (IQR: 77–97), 83 (IQR: 70–93), and 83.5 (IQR: 76–97) at 18 months (corrected age), 3 years, and 6 years, respectively.

**Table 1:** The perinatal demographic characteristics.

	Overall (*n* = 218)	Survival cases (*n* = 198)	DQ at 18 months (corrected age) is available (*n* = 159)	DQ at 3 years is available (*n* = 144)	IQ at 6 years is available (*n* = 100)
Females/Males	107/111	97/101	79/80	67/77	55/45
Gestational age (weeks) †	26 (25–27)	26 (25–27)	27 (25–28)	26 (25–27)	26.5 (25–28)
Birth weight (g) †	757 (592–908)	778 (617–919)	794 (622–912.5)	781.5 (630–912)	800.5 (608–929)
Apgar score (5 min) †	6 (4–8)	6 (4–8)	6 (4.5–8)	6 (5–8)	6 (5–8)
Singleton	167 (77%)	149 (75%)	116 (73%)	106 (74%)	75 (75%)
Antenatal steroid	142 (66%)	133 (68%)	109 (69%)	100 (70%)	65 (65%)
Pre-labor rupture of membranes	69 (32%)	62 (32%)	48 (31%)	47 (33%)	31 (31%)
Cesarean delivery	180 (85%)	169 (86%)	134 (84%)	109 (83%)	87 (87%)
Hydrocortisone use	113 (52%)	109 (55%)	82 (52%)	76 (53%)	55 (55%)
Dexamethasone use	5 (2%)	4 (2%)	3 (2%)	2 (1%)	2 (2%)
Sepsis	17 (8%)	13 (7%)	8 (5%)	5 (3%)	3 (3%)
NEC (grade ≥II)	7 (3%)	4 (2%)	3 (2%)	3 (2%)	1 (1%)
IVH (grade ≥III)	18 (9%)	13 (7%)	11 (7%)	10 (7%)	4 (4%)
PVL	9 (4%)	9 (5%)	3 (2%)	2 (1%)	1 (1%)
BPD	128 (64%)	122 (64%)	95 (62%)	86 (61%)	65 (65%)
LCC	57 (28%)	55 (28%)	40 (25%)	37 (26%)	26 (26%)
DQ/IQ score †			88 (77–97)	83 (70–93)	83.5 (76–97)

*DQ*developmental quotient,*IQ*intelligence quotient, *IVH* intraventricular hemorrhage, *PVL* periventricular leukomalacia; *BPD* bronchopulmonary dysplasia, *NEC* necrotizing enterocolitis, *LCC* late-onset circulatory collapse.

†Median (IQR).

**Fig. 1:**
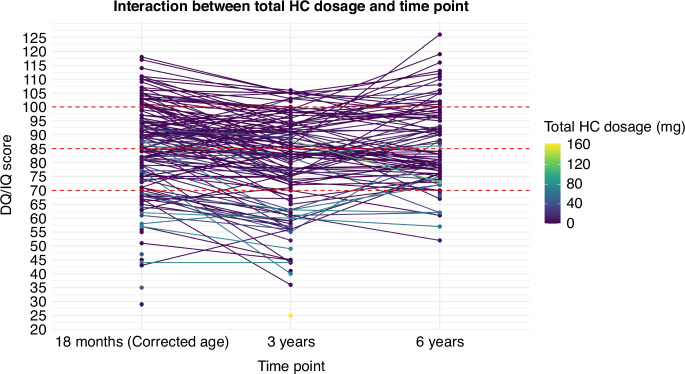
Interactions between total hydrocortisone dosages administered to infants with extremely low birth weights and their DQ/IQ scores over the first six years. DQ, developmental quotient; HC, hydrocortisone; IQ, intelligence quotient.

Interaction plots showing the DQ/IQ trajectories of our cohort at 18 months (corrected age), 3 years, and 6 years are presented in Fig. 1. In the figure, higher HC doses (light blue, green, and yellow) are more apparent in infants with DQ/IQ scores <85. This trend becomes more pronounced in those with DQ/IQ scores <70. In children with particularly low initial DQ scores, subsequent DQ/IQ results were lacking in many cases.

Supplemental Table S1 presents cases in which IQ testing was not performed at 6 years of age, grouped according to the reason for the absence of this data. Among the patients followed up, 20 could not be tested because of severe neurodevelopmental disorders, cerebral palsy, or developmental disorders. Many of these patients also did not have developmental examinations at the other two time points. The total dose of HC tended to be higher in these cases than in the children who underwent IQ testing. As shown in Supplemental Table S1, the median gestational age in this group was 25 weeks (IQR: 24–27), which is lower than that of children who completed the IQ assessment.

### Assessment of linear model fit using LOESS

A nonlinear LOESS trend analysis was conducted to evaluate the appropriateness of a linear model for the main analysis (Supplemental Fig. S2). The LOESS plots indicated that DQ/IQ scores tended to decrease with increasing cumulative HC dosage across all age groups, suggesting a dose-dependent relationship. A non-linear pattern was observed at approximately 20 mg. However, this was likely attributable to overfitting caused by a high density of data points around this dosage rather than representing a true non-linear effect. Overall, the results supported the feasibility of a linear model for subsequent analyses. At lower dosages, around 10 mg, the impact of HC on DQ/IQ appeared negligible. Nevertheless, these preliminary findings should be interpreted cautiously as they did not account for potential confounding factors.

### Linear mixed model analysis and longitudinal trends over time

**Fig. 2:**
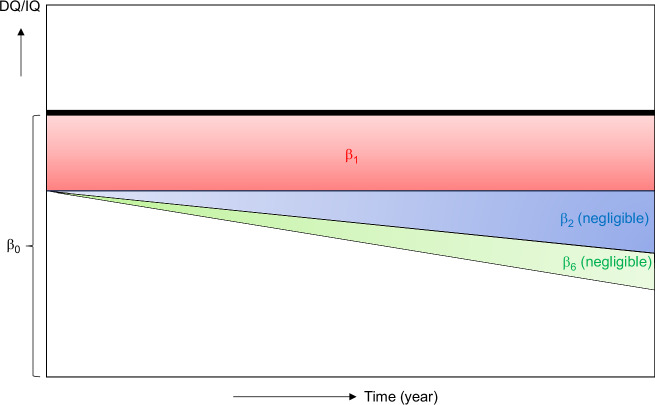
Results of a linear mixed model analysis of the relationship between total hydrocortisone dosages administered to infants with extremely low birth weights and their DQ/IQ scores over the first six years. The impact of each clinical and demographic variable on the DQ/IQ over time is shown. β0 : The average DQ/IQ at baseline, which was 88.65 in the adjusted model (95% CI: 84.65 – 92.74, *p *< 0.001). β1 : The change in DQ/IQ due to HC administration, which was −2.82 per 10 mg of HC in the adjusted model (95% CI: −3.89 to −1.06, *p* = 0.001). β2 : The change in DQ/IQ over time, which was −0.57 per time point in the adjusted model (95% CI: −1.19 – 0.06, *p* = 0.075) β6 : The change in DQ/IQ over time when HC is administered, which was 0.10 in the adjusted model (95% CI: −0.18 – 0.37, *p* = 0.481). DQ, developmental quotient; HC, hydrocortisone; IQ, intelligence quotient.

**Fig. 3:**
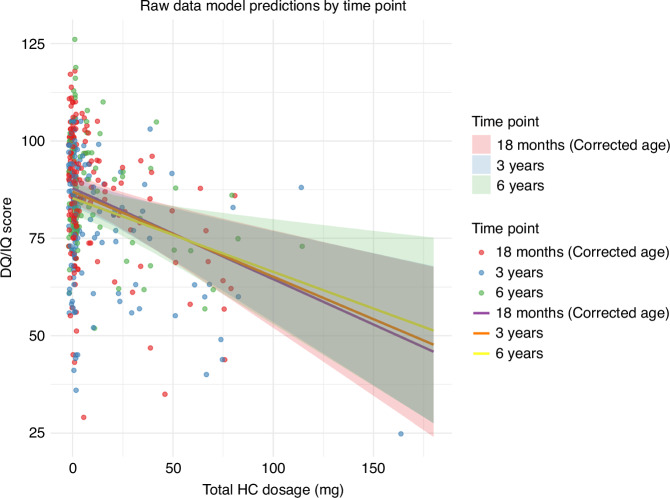
Raw data and model predictions for analysis of the relationship between total hydrocortisone dosages administered to infants with extremely low birth weights and their DQ/IQ scores for three different time points. DQ, developmental quotient; HC, hydrocortisone; IQ, intelligence quotient.

Figure 2 and Table 2 present the results of our LMM analysis. Again, we found that higher HC dosages were significantly related to lower DQ/IQ scores. This trend persisted at 6 years of age, suggesting a sustained effect of HC on cognitive outcomes. The adjusted model estimated an average DQ/IQ of 88.65 at baseline (95% CI: 84.65–92.74, *p* < 0.001). For every 10 mg increase in HC dosage, IQ scores were estimated to decrease by 2.82 points (95% CI: −3.89 to −1.06, *p* = 0.001). The overall reduction in DQ/IQ over the time trajectory was minimal, with a non-significant decrease of 0.57 points per year (95% CI: −1.19 to 0.06, *p* = 0.075), indicating that temporal changes in DQ/IQ scores were small and not strongly influenced by the time factor itself. The interaction term between HC dosage and time was not statistically significant (0.10, 95% CI: −0.18 to 0.37, *p* = 0.481), suggesting that the effect of HC dosage on DQ/IQ did not vary substantially throughout the study period. As described in the Methods section, we also ran sensitivity models replacing the composite “severe complications” variable with either birth weight or gestational weeks. These models yielded similar findings. Of note, birth weight was a significant predictor of DQ/IQ scores, while gestational weeks was not (Supplemental Table S2). The marginal effects of HC dosage and time point were plotted in Fig. 3 as a visual representation of these results. As can be seen in the figure, there was a consistent decline in DQ/IQ scores with increasing HC dosage across all three time points (18 months (corrected age), 3 years, and 6 years). The slopes for each time point are nearly parallel, illustrating that the interaction between HC dosage and time was minimal.

**Table 2:** The results of the Linear Mixed Model analysis.

Linear Mixed Model Results						
	Non-Adjusted Model			Adjusted Model		
*Predictors*	*Estimates*	*CI*	*p*	*Estimates*	*CI*	*p*
(Intercept)	86.99	83.85 – 90.13		88.65	84.65 – 92.74	
Total HC dosage per 10 mg	−3.17	−4.48 – −1.86	<0.001	−2.82	−3.89 – −1.06	0.001
Time point	−0.57	−1.20 – 0.05	0.073	−0.57	−1.19 – 0.06	0.075
Total HC dosage per 10 mg: Time point	0.14	−0.14 – 0.41	0.334	0.10	−0.18 – 0.37	0.481
Severe complications: Yes (Sepsis, NEC (grades ≥ II), IVH (grades ≥ III), PVL: Any one of these yes)				−13.58	−19.84 – −7.31	<0.001
BPD: Yes				1.09	−3.44 – 5.62	0.637
LCC: Yes				−4.28	−9.89 – 1.34	0.135
Random Effects						
σ^2^	85.45			85.37		
τ_00_	178.26 _Case number_			156.77 _Case number_		
*N*	165 _Case number_			165 _Case number_		
Observations	396			396		

*HC* hydrocortisone, *NEC* necrotizing enterocolitis, *IVH* intraventricular hemorrhage, *PVL* periventricular leukomalacia, *BPD* bronchopulmonary dysplasia, *LCC* late-onset circulatory collapse.

## Discussion

Most studies to date have found correlations between neonatal steroid treatment and subsequent neurodevelopmental outcomes at a single later time point. The current inaugural study investigated the relationship between the total doses of HC administered to ELBW infants during the neonatal period and their DQ/IQ trajectory up to school age. We found an association between total HC dosage and DQ/IQ over time. After adjusting for potential confounding factors such as the presence or absence of severe complications such as sepsis, necrotizing enterocolitis, intracranial hemorrhage, and periventricular leukomalacia; and the presence or absence of BPD or LCC, which are often treated with HC, this relationship persisted.

This study showed an association between total neonatal HC dosage for ELBW infants and DQ/IQ until school age, with the effect persisting over time. Previous studies on this topic have obtained different results, with no association between the administration of HC to infants with very low birth weight and their later neurodevelopmental outcomes.^17–22^ However, none of the studies that reported any significant effect accounted for the total HC dosage administered during the neonatal period. This may explain why their results differed from ours and those of other authors.

It is well established that dexamethasone negatively affects neonatal neurodevelopment.^8–10^ Previous reports have highlighted differences in glucocorticoid activity, half-life, and receptor affinities between dexamethasone and HC, and these factors could cause the two types of steroids to differ in their effects on neonatal neurodevelopment.^15,31,32^ However, the findings of the present study suggest that, despite these differences, as glucocorticoids, both dexamethasone and HC affect neonatal neurodevelopment, albeit to varying extents. The underlying mechanisms by which perinatal glucocorticoid exposure exerts its long-term effects are not yet fully understood. Previous animal studies have demonstrated that both dexamethasone and HC can transiently suppress neuronal proliferation and astrogliosis.^33,34^ Moreover, the administration of exogenous glucocorticoids has been associated with adverse neuropsychiatric effects, manifesting not only as severe mood disturbances such as depression and mania, but also as cognitive impairments, including deficits in concentration and memory.^35^ It is plausible that brain function may be adversely affected by glucocorticoid administration during the neonatal period. Currently, HC is administered to infants with ELBW for various reasons, including BPD prevention and treatment, management of hypotension, and treatment of LCC. HC plays an integral role in the respiratory and circulatory management of ELBW infants. As such, it could be argued that neurodevelopmental outcomes could be more adversely affected by failure to administer HC in these situations. Because of the risks involved in both the administration and non-administration of HC, it is ethically unfeasible to assess the effects of HC on ELBW infants with a fully randomized trial. Therefore, in this study, we were only able to evaluate the relationship between HC dosage and subsequent neurodevelopmental outcomes retrospectively. In our analysis, gestational age was not a significant predictor of DQ/IQ scores in the multivariable model. This finding contrasts with previous large-scale study, which reported a strong association between lower gestational age and poorer cognitive outcomes in extremely preterm infants.^36^ One possible explanation is that our inclusion criteria were based on birth weight <1000 g, regardless of gestational age. As a result, our cohort likely included infants with more advanced gestational age but severe intrauterine growth restriction. These infants may have distinct developmental trajectories, which could have attenuated the observed effect of gestational age in our analysis. This characteristic of our cohort may also partly explain another finding: the incidence of late-onset circulatory collapse (LCC) appeared to be somewhat higher than previously reported in extremely low birth weight infants. For example, a prior study reported an incidence of 13.4% among infants with birth weight <1000 g.^37^ Although the reason for this difference remains unclear, it may reflect population-specific characteristics or institutional practices.

This investigation had some limitations. First, the retrospective design of the study introduced the possibility of bias from unrecognized confounding factors. However, we made efforts to minimize this bias as much as possible by statistically controlling for known confounders related to HC administration and developmental outcomes. An association was found between total HC dosage and later DQ/IQ, even after considering these potential confounders. Additionally, we were unable to evaluate several important potential confounding factors, such as nutritional status during NICU hospitalization, access to early intervention services, and socioeconomic status, due to a lack of data consistency across centers. This limits our ability to fully account for their influence on neurodevelopmental outcomes. Furthermore, the relatively small sample size, despite being a multicenter study, may limit the generalizability of our findings to broader populations. A further limitation was the length of the follow-up period, which inevitably increased the dropout rate and the amount of missing data. A previous randomized controlled trial that followed ELBW infants for the first five years reported that only about 75% of surviving cases were evaluable at 5 years of age, with the other 25% lost to follow-up.^22^ In the present study, 135 (68%) of the 198 surviving cases were followed-up for the full 6 years. This is roughly comparable to the follow-up rates reported in previous studies. Nonetheless, it should be noted that a certain degree of data loss occurred and this may have affected our findings. A LMM analysis was selected to compensate for this because it is known to be relatively robust to missing data.^38^ Despite its retrospective nature, a key strength of this investigation was the application of statistical techniques aimed at mitigating its inherent limitations. Finally, although the study was conducted across multiple centers, center-level effects were not included in the statistical models due to the small sample size and uneven distribution of participants per site. This may have introduced unmeasured center-related variability that could influence the precision of the estimated associations.

Longitudinal studies investigating outcomes that emerge months or years after the exposure face intrinsic methodological challenges, particularly when events along the timeline may lie on the causal pathway. In our study, variables such as severe complications could be considered mediators rather than confounders, as they may be influenced by the exposure (hydrocortisone) and in turn affect neurodevelopmental outcomes. While we included them as covariates to control for clinical severity, we recognize that this approach may partially adjust away part of the exposure effect. This highlights the complexity of analyzing long-term outcomes in neonatal cohorts and underlines the importance of interpreting adjusted models within their causal framework.

The central finding of this study was that a higher total dose of HC administered up to 36 weeks PMA to ELBW infants is associated with persistent lower DQ/IQ scores. This suggests that, while HC is essential to the respiratory and circulatory management of infants with ELBW, the benefits of higher doses of HC during treatment should be carefully weighed against the detrimental effects of high doses on neurodevelopmental outcomes.

## Conclusions

This study found a relationship between higher total doses of HC administered to ELBW infants up to 36 weeks PMA and impaired developmental outcomes that persisted at school age. Although HC is commonly used in the management of these infants, clinicians should be aware of the potential dose-dependent effect of HC on neurodevelopmental outcomes.

## Supplementary information

Supplementary Information

## Data Availability

Data are available on reasonable request. The deidentified participant data and study protocol are available to investigators whose proposed use of the data has been approved by an independent ethics review committee for this purpose.
