# Supplementary material for: Total hydrocortisone dosage in extremely low birth weight infants and neurodevelopment up to school age
Source: Pediatr Res. 2025 Sep 23;99(4):1451–7. doi: 10.1038/s41390-025-04426-x (PMC13102704; doi:10.1038/s41390-025-04426-x)

Supplemental Table S1. Classification of cases where IQ testing was not available at 6 years old, according to the reason for the absence.

	N	Able<br><br>N = 100	Unable (Drop-out/ Physician changed)<br>N = 63	Unable (Cerebral palsy/ Developmental disability)<br>N = 20	Unable (No test desired or other tests conducted)<br>N = 15
**Birth Hospital**	198				
Hospital A		29 (29%)	15 (24%)	5 (25%)	4 (27%)
Hospital B		5 (5.0%)	3 (4.8%)	2 (10%)	0 (0%)
Hospital C		8 (8.0%)	6 (9.5%)	1 (5.0%)	0 (0%)
Hospital D		35 (35%)	25 (40%)	10 (50%)	8 (53%)
Hospital E		7 (7.0%)	7 (11%)	0 (0%)	1 (6.7%)
Hospital F		10 (10%)	4 (6.3%)	0 (0%)	0 (0%)
Hospital G		4 (4.0%)	0 (0%)	0 (0%)	2 (13%)
Hospital H		2 (2.0%)	3 (4.8%)	2 (10%)	0 (0%)
**Birth Weight (g) †**	198	801 (613–928)	799 (672–934)	695 (520–807)	708 (552–875)
**Gestational Age (weeks)†**	198	27 (25–28)	26 (25–27)	25 (24–27)	26 (24–29)
**Dexamethasone use**	198	2 (2.0%)	1 (1.6%)	0 (0%)	1 (6.7%)
**Sepsis**	198	3 (3.0%)	7 (11%)	2 (10%)	1 (6.7%)
**NEC (grades ≥II)**	198	1 (1.0%)	2 (3.2%)	0 (0%)	1 (6.7%)
**IVH (grades ≥III)**	198	4 (4.0%)	3 (4.8%)	5 (25%)	1 (6.7%)
**PVL**	198	1 (1.0%)	3 (4.8%)	5 (25%)	0 (0%)

	N	Able<br><br>N = 100	Unable (Drop-out/ Physician changed)<br>N = 63	Unable (Cerebral palsy/ Developmental disability)<br>N = 20	Unable (No test desired or other tests conducted)<br>N = 15
**BPD**	198	65 (65%)	37 (59%)	12 (60%)	8 (53%)
**LCC**	198	26 (26%)	13 (21%)	11 (55%)	5 (33%)
**DQ Score at 18 months (corrected age) †**	159	90 (82–101)	90 (79–99)	64 (48–83)	77 (68–85)
Unknown		5	30	4	0
**DQ Score at 3 years†**	144	86 (77–94)	85 (70–95)	51 (44–68)	72 (57–89)
Unknown		8	39	6	1
**IQ score at 6 years†**	100	84 (76–97)	NA	NA	NA
Unknown		0	63	20	15
**Total HC dosage (mg) †**	198	0.11 (0.00–1.08)	0.07 (0.00–0.64)	0.82 (0.00–5.97)	0.12 (0.00–1.40)

†Median (IQR)

NEC, necrotizing enterocolitis; IVH, intraventricular hemorrhage; PVL, periventricular leukomalacia; BPD, bronchopulmonary dysplasia; LCC, late-onset circulatory collapse; DQ, developmental quotient; IQ, intelligence quotient; HC, hydrocortisone

Supplemental Table S2. The results of the Linear Mixed Model analysis.

Linear Mixed Model Results

	**Adjusted Model 1**			**Adjusted Model 2**		
Predictors	*Estimates*	*CI*	*p*	*Estimates*	*CI*	*p*
**(Intercept)**	73.13	60.25 – 86.00		97.47	64.60 – 130.33	
**Total HC dosage per 10mg**	−2.81	−4.25 – −1.37	**<0.001**	−2.88	−4.35 – −1.42	**<0.001**
**Time point**	−0.57	−1.19 – 0.06	0.076	−0.57	−1.19 – 0.06	0.076
**Total HC dosage per 10mg: Time point**	0.13	−0.14 – 0.41	0.347	0.13	−0.15 – 0.40	0.357
**Birth weight (per100g)**	1.61	0.17 – 3.05	**0.028**			
**Gestational weeks**				−0.39	−1.58 – 0.80	0.521
**BPD: Yes**	2.62	−2.18 – 7.42	0.283	1.26	−3.60 – 6.12	0.610
**LCC: Yes**	−1.76	−8.09 – 4.58	0.586	−4.95	−11.02 – 1.13	0.110
**Random Effects**						
**σ^2**	85.41			85.31		
**τ_{00}**	172.49 Case number			178.74 Case number		
**N**	165 Case number			165 Case number		
**Observations**	396			396		

HC, hydrocortisone; NEC, necrotizing enterocolitis; IVH, intraventricular hemorrhage; PVL, periventricular leukomalacia; BPD, bronchopulmonary dysplasia; LCC, late-onset circulatory collapse;

Supplemental Figure S1.

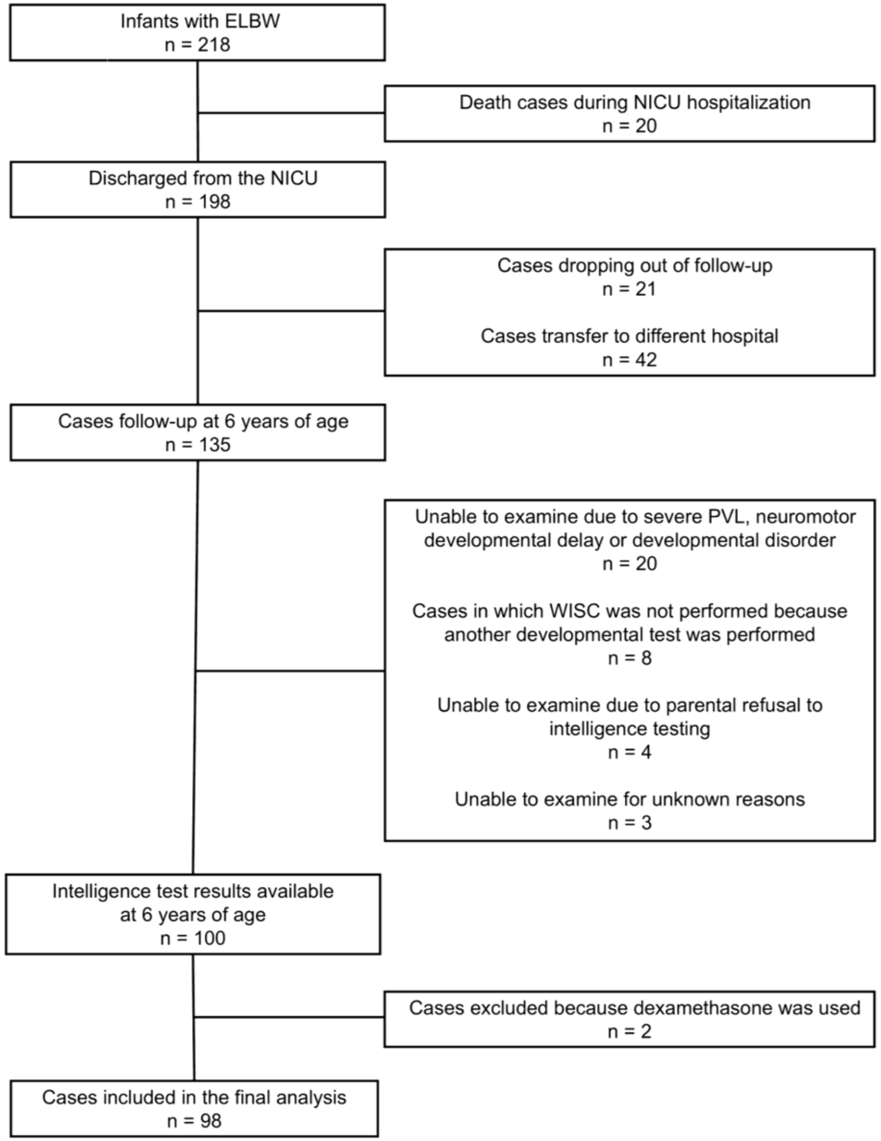
Infants with ELBW
n = 218
Death cases during NICU hospitalization
n = 20
Discharged from the NICU
n = 198
Cases dropping out of follow-up
n = 21
Cases transfer to different hospital
n = 42
Cases follow-up at 6 years of age
n = 135
Unable to examine due to severe PVL, neuromotor developmental delay or developmental disorder
n = 20
Cases in which WISC was not performed because another developmental test was performed
n = 8
Unable to examine due to parental refusal to intelligence testing
n = 4
Unable to examine for unknown reasons
n = 3
Intelligence test results available at 6 years of age
n = 100
Cases excluded because dexamethasone was used
n = 2
Cases included in the final analysis
n = 98

Supplemental Figure S2.

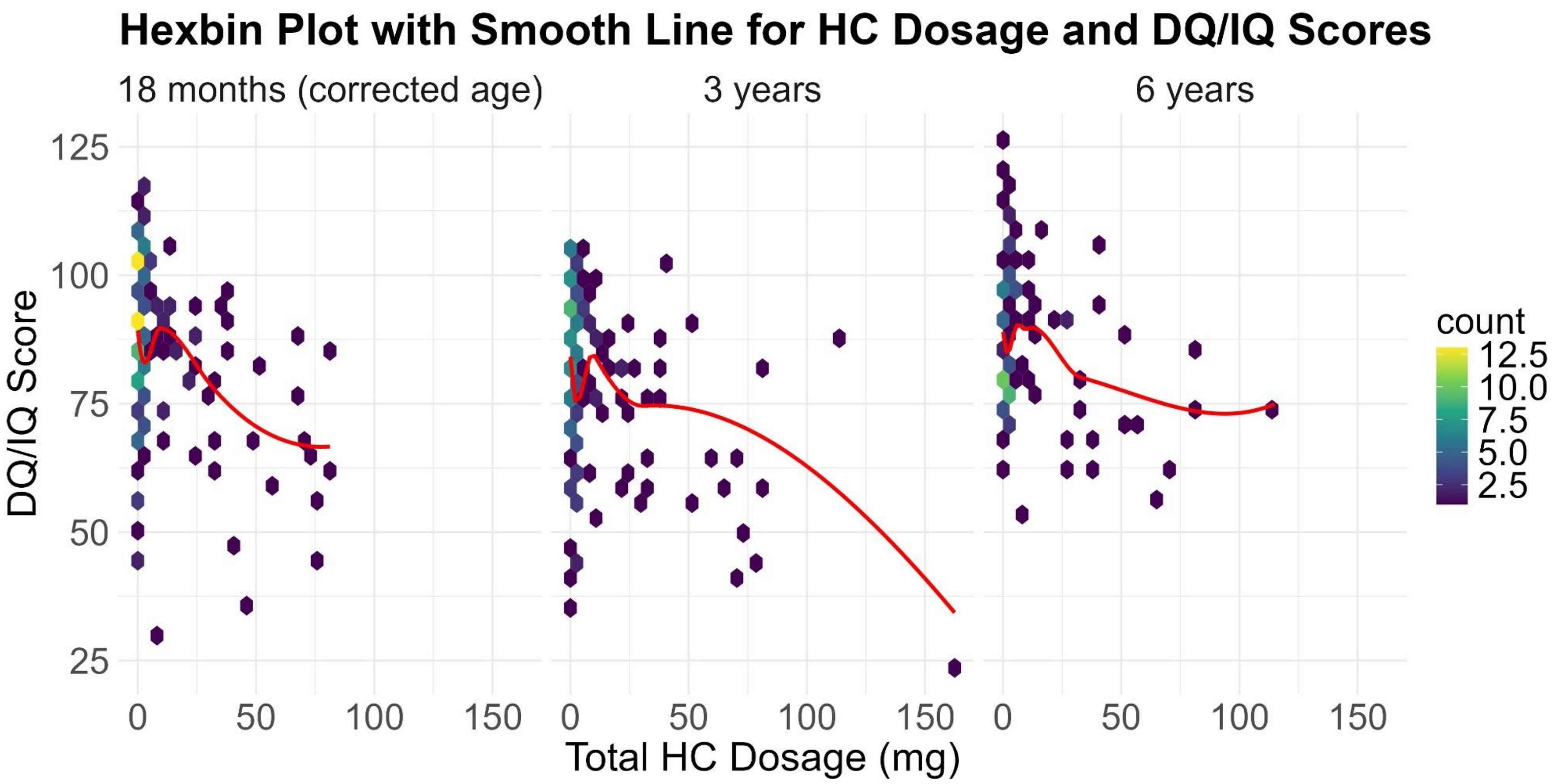

Supplement: Supplementary file 1 — Supplementary Information [file 41390_2025_4426_MOESM1_ESM.pdf]
